# Electrochemotherapy for basal cell carcinoma in the head and neck region: 5-year follow-up from the Insp-ECT registry

**DOI:** 10.2478/raon-2025-0040

**Published:** 2025-06-21

**Authors:** Ales Groselj, Giulia Bertino, Marta Minuti, Anthony James P Clover, Camilla Kjaer Lonkvist, Erika Kis, Christian Kunte, Tobian Muir, Francesco Russano, Francesca de Terlizzi, Joy Odili, Gregor Sersa

**Affiliations:** 1 Department of Otorhinolaryngology and Cervicofacial Surgery, University Medical Centre Ljubljana, Ljubljana, Slovenia; 2 Department of Otolaryngology-Head and Neck Surgery, IRCCS Policlinico San Matteo Foundation, Pavia, Italy; 3 Department of Plastic Surgery, Cork University Hospital and Cancer Research@UCC, University College Cork; 4 Department of Oncology, Herlev and Gentofte Hospital, University of Copenhagen, Herlev, Denmark; 5 Department of Dermatology and Allergology, Medical University of Szeged, Szeged, Hungary; 6 Department of Dermatologic surgery and Dermatology, Artemed Fachklinik München, Munich, Germany; 7 Department of Dermatology and Allergology, Ludwig-Maximilian University Munich, Munich, Germany; 8 Plastic and Reconstructive Surgery Department, James Cook University Hospital, Marton Road, Middlesbrough, United Kingdom; 9 Soft-Tissue, Peritoneum and Melanoma Surgical Oncology Unit, Veneto Institute of Oncology IOV-IRCCS, Padua, Italy; 10 IGEA Biophysics Lab. Carpi, Modena, Italy; 11 St George’s University Hospital NHS Foundation Trust, London, United Kingdom; 12 Department of Experimental Oncology, Institute of Oncology Ljubljana, Ljubljana, Slovenia; 13 Faculty of Health Sciences, University of Ljubljana, Ljubljana, Slovenia

**Keywords:** basal cell carcinoma, electrochemotherapy, recurrence free survival, 5 years

## Abstract

**Background:**

Basal cell carcinoma (BCC) is a cutaneous malignancy that typically appears in sun-exposed areas. We analyzed data from the Insp-ECT registry of all patients affected by BCC in the head and neck region. The aim of this study was to evaluate the safety and efficacy of electrochemotherapy (ECT) on a 5-year basis.

**Patients and methods:**

A cohort of 132 patients was included. They were treated by ECT according to the current Standard Operating Procedures. The median age was 74 years (range 41–93). There was a median of 1 nodule per patient (range 1–7), and the median size of the lesions was 1.4 cm (range 0.5–5.0 cm).

**Results:**

Patients tolerated ECT well, and 96% agreed to repeat it if needed. The side effects were mild and temporary. All patients achieved a complete clinical response after 1 to 3 ECT sessions. During the first year of follow-up, 4 (3%) patients experienced recurrence, which was treated (2 with ECT, 1 with surgery, and 1 with a combination of ECT and surgery), after which they remained free of disease until the end of follow-up at 5 years. Five patients reported recurrence thereafter and were treated according to their condition. At the 5-year follow-up, the disease-free survival (DFS) rate was 92% (95% confidence interval [CI]: 87%–96%). At that time, 3 patients were alive with disease (2%), and 124 patients were free of disease (98%).

**Conclusions:**

This study shows the feasibility and efficacy of ECT treatment in elderly patients with BCC tumors in aesthetically and functionally sensitive areas, with negligible toxicity. Comparable efficacy to other treatment modalities was demonstrated at 1 year and 5 years of follow-up in terms of DFS.

## Introduction

Basal cell carcinoma (BCC) is the most common malignant tumor among the white population, with a globally increasing incidence.^1^ Individuals with Fitzpatrick skin types I and II have a greater risk of developing BCC, with an estimated lifetime risk of 30%. The likelihood of BCC is also increased in people with light eye color, freckles, and blonde or red hair.^2^ UV radiation exposure is the most significant environmental risk factor; thus, BCC typically appears on sun-exposed areas of the skin. Other risk factors include childhood sunburns, a family history of skin cancer, tanning bed use, chronic immunosuppression, photosensitizing drugs, ionizing radiation, and exposure to carcinogenic chemicals, particularly arsenic. Childhood sun exposure and intense, intermittent sun exposure are strongly linked to BCC development. This explains why approximately 80% of BCCs occur in the head and neck region.^3^

The standard treatments for BCC are surgical excision and Mohs micrographic surgery.^4^ Surgical excision is typically used for low-risk lesions located in easily accessible areas, whereas Mohs surgery is recommended for high-risk lesions.^4^ The five-year recurrence rate is lower for Mohs surgery than for standard excision for both primary and recurrent BCCs, at 1% and 6%, respectively.^4^ The destructive nature of BCC can result in significant physical and psychological morbidity after treatment, as many lesions develop in functionally and aesthetically important areas. Other local treatment options include cryosurgery, electrodesiccation and curettage, topical application of imiquimod or fluorouracil, photodynamic therapy, and radiation therapy.^5^ The choice of treatment depends on the patient’s condition, tumor location, and risk of recurrence.^6^

Radical surgical treatment or radiotherapy is not always the best treatment, especially for locally advanced BCC, which affects functionally and aesthetically important areas.^7^ Particularly in the elderly population, systemic targeted therapy or immunotherapy may not always be feasible due to comorbidities. In such cases, local treatments such as electrochemotherapy (ECT) may be considered.^8–10^ ECT emerged in the late 1990s in Europe as a highly effective, minimally invasive local therapy for skin cancer treatment.^11^ Recent studies reported clearance rates ranging from 85% to 96% in BCCs treated with ECT.^12–15^

The technique involves intravenous (IV) or intratumoral (IT) administration of a cytotoxic agent, primary cisplatin or bleomycin, combined with electroporation. During electroporation, short high-voltage electric pulses transiently permea-bilize tumor cell membranes, allowing cytotoxic agents to diffuse into the cells more effectively, thereby increasing their cytotoxicity.^16^

ECT has been proven to be a safe and effective treatment for various types of solid skin tumors. Its application is well established for treating cutaneous and subcutaneous nodules, regardless of the tumor’s histology.^13,14,17,18^ Most importantly, the mechanisms of its action are well understood. In addition to directly killing tumor cells, it also has local immunological effects due to immunogenic tumor cell death and shedding of tumor antigens.^19^ Furthermore, it exerts an indirect effect on the tumor vasculature. Electroporation itself triggers vasoconstriction, leading to transient ischemia. This is the so-called vascular lock effect, which in ECT delays the early washout of the cytotoxic drug from the tumor. However, bleomycin is also cytotoxic to endothelial cells, which later induces a vascular disrupting effect. The vascular disrupting effect is selective for the tumor vasculature but preserves the adjacent normal tissue vasculature. Considering these effects, ECT represents an effective ablative therapy because of its targeted and selective mechanisms.^20,21^

The recently published European guidelines on the diagnosis and treatment of BCC highlight the important role of ECT as a treatment option.^6^ It may be considered when surgery or radiotherapy is not feasible or contraindicated, in cases of lesions in critical areas (central face, eyelid, eyebrow, nose, lips, chin, ear, periauricular), poorly defined margins, recurrent BCC, aggressive subtypes, perineural invasion, or multiple lesions.^6^ ECT may also be considered an appropriate treatment option for BCC recurrences following standard treatments.^15^

The primary aim of the present study was to analyze patients with BCC in the head and neck region treated with ECT from the Insp-ECT database and to elucidate long-term outcomes over 5 years. The secondary aim was to assess the safety and tolerability of ECT in the elderly population of patients.

## Patients and methods

### Patients and database

The data for this study were obtained from the Insp-ECT database. The Insp-ECT registry is a prospective database established in 2008 to assess the outcome of patients with skin cancers or cutaneous metastases treated with ECT (https://insp-ect.eu) according to the published Standard Operating Procedure.^22,23^

The study was conducted in accordance with the principles of Good Clinical Practice and tenets of the Declaration of Helsinki. The collected information included patient demographics, ECT parameters, tumor characteristics, tumor response, treatment toxicity and patient-reported outcomes, as well as potential later treatments. Written informed consent was obtained from each patient, and approval from the ethics committee and data protection authority was sought by each institution. The inclusion and exclusion criteria followed the Standard Operating Procedures for ECT.^22,23^ The preoperative workup included a complete medical history, physical examination, radiological examination when necessary, and standard blood tests.

For the data analysis, eligible patients were selected on the basis of the presence of primary or recurrent BCC for which other treatment modalities had failed or were inapplicable. In particular, ECT treatment was considered for carefully selected patients affected by BCC located in critical areas (central face, eyelid, eyebrow, nose, lips, chin, ear, and periauricular regions) in the following cases:

a) primary lesions where all other treatment options, including surgery and radiotherapy, were contraindicated due to radically unresectable disease, a high risk of functional organ damage or because of a precarious physical condition resulting from age and comorbidities;
b) persistent or recurrent primary BCC lesions when all other treatment options, including surgery and radiotherapy, failed or were not feasible;
(c) when the patient, after being comprehensively informed, declined all other treatment options.

The primary aim of the present study was to analyze patients with BCC in the head and neck region treated with ECT from the Insp-ECT database and to elucidate long-term outcomes over 5 years. The secondary aim was to assess the safety and tolerability of ECT in the elderly population of patients.

### Procedure

**FIGURE 1.**
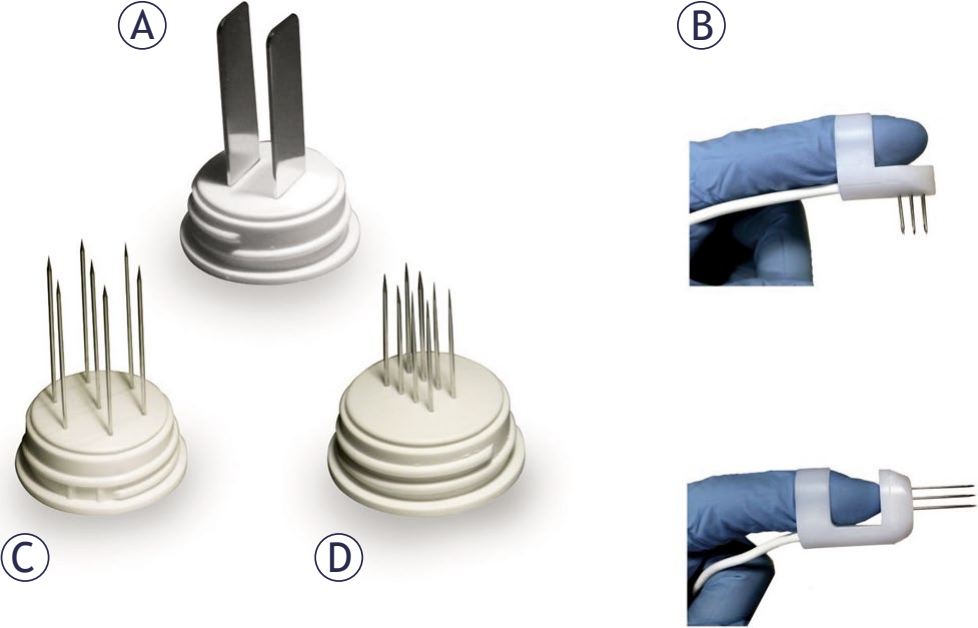
Types of electrodes used. **(A)** Type I: two plates with a 6 mm gap; **(B)** type IV: a wearable finger electrode with two parallel rows of needles; **(C)** type III: a hexagonal array with a 7.3 mm gap; or **(D)** type II: two parallel rows of needles with a 4 mm gap.

ECT was performed according to the European Standard Operating Procedures on Electrochemotherapy.^22,23^ Briefly, bleomycin was administered either intratumoral (IT) (max 1,000 IU/cm^3^ lesion volume) or intravenous (IV) (15,000 IU/m^2^ body surface area), depending on the number and size of the tumors. The choice of electrode for ECT was based on the site and size of the lesion (Figure 1). Eight electrical pulses of 100 μs duration were delivered using Cliniporator, a squarewave electric pulse generator (IGEA, Carpi, Italy).

Tumor response was evaluated according to the Response Evaluation Criteria in Solid Tumors (RECIST version 1.0).^24^ The response of each target lesion was assessed at follow-up visits conducted at 1, 2, 6, and 12 months following ECT, with subsequent longitudinal monitoring of all patients to evaluate long-term outcomes.

Pain intensity was evaluated using a visual numeric scale (VNS). Tumor-related symptoms were registered following the CTC 4.0 criteria. The specific analysis considered ulcerations, hyperpigmentation, bleeding and other local effects such as rash, itching and oedema of the treated area.

### Statistical analysis

Descriptive analysis included the median and range and the mean and standard deviation for continuous numeric variables. Comparisons between groups were performed by 2-tailed heteroscedastic uncoupled t test, whereas comparisons of repeated measurements in the same group over time were performed via a 2-tailed coupled Student’s t test for continuous variables. Categorical variables are reported as absolute counts and percentages. Comparisons between categorical variables were performed via contingency table analysis and the Chi-square test with correction for repeated measurements. Disease-free survival (DFS) was defined as the time from the first ECT up to the date of relapse or the last follow-up. Survival curves for DFS were generated via the Kaplan–Meier method. NCSS 9 (NCSS, LLC. Kaysville, Utah, USA; www.ncss.com) software was used for the analysis.

## Results

A total of 156 patients with BCC who were treated with ECT more than 5 years ago were identified in the Insp-ECT database. Patients were enrolled in 9 Insp-ECT centers: Pavia, Szeged, Ljubljana, Middlesbrough, London, Padova, München, Cork and Herlev. Among the entire patient cohort, 132 (85%) successfully completed at least 5 years of follow-up. The 24 remaining patients were excluded from the analysis, of whom 5 were lost to followup and 19 died due to causes unrelated to the study. The median follow-up time for the 132 included patients was 5.8 years (range 5.1–11.6 years). In this cohort, the median age was 74 years (range 41–93 years, mean 72.7 ± 10.8 years). Among them, 98 patients (74%) were older than 65 years, whereas 34 (26%) were younger than 65 years. The descriptive characteristics of the population are reported in Table 1.

**TABLE 1.** Descriptive characteristics of the population

	N	%
GENDER		
Males	80	61%
Female	52	39%
LESIONS’ NUMEROSITY		
Single	102	77%
Multiple	30	23%
T STAGE		
T1	99	75%
T2	11	8%
T3	1	1%
Tis	21	16%
TYPE OF TUMOR		
Primary	72	55%
Recurrent	60	45%
ULCERATED		
No	96	73%
Yes	36	27%
LESIONS’ PREIRRADIATION		
Yes	3	2%
PREVIOUS TREATMENTS		
Surgery	52	39%
Radiotherapy	2	2%
Surgery, radiotherapy	6	5%
Surgery, other therapies	1	1%
Surgery, radiotherapy, other therapies	2	2%
No	69	51%

One or a few lesions up to 5 cm in diameter were treated with either local or systemic bleomycin administration. Most patients (77%) had a single nodule. The median number of nodules per patient was 1 (range 1–7, mean 2.1 ± 1.8), and the median size of the lesions was 1.4 cm (range 0.5– 5.0 cm, mean 1.5 ± 1.3 cm). A total of 158 target nodules from 132 patients were analyzed. The nodules were predominantly located in aesthetically and functionally sensitive areas, as shown in Table 2.

**TABLE 2.** Locations of nodules

LOCALIZATION	N	%
Nose	65	41%
Ear	20	12%
Forehead/temple	16	10%
Eye	16	10%
Cheek	6	4%
Scalp	5	3%
Lip	4	3%
Chin	3	2%
Neck	1	1%
Other locations	22	14%

ECT treatment was conducted under local anesthesia and deep sedation in 100 patients (76%) and under general anesthesia in 32 patients (24%). Bleomycin was administered systemically (IV) in 76 patients (58%) and locally (IT) in 56 patients (42%). Among the 100 patients treated under local anesthesia, approximately half of them received IT and the other half IV bleomycin administration (Table 3). Among the 32 patients treated under general anesthesia, dominated IV drug administration (Table 3).

**TABLE 3.** Distribution of patients according to drug delivery and type of anesthesia

	Local drug (IT)	Systemic drug (IV)
Local anesthesia	47	53
General anesthesia	9	23

1 IT = intratumoral; IV = intravenous

**FIGURE 2.**
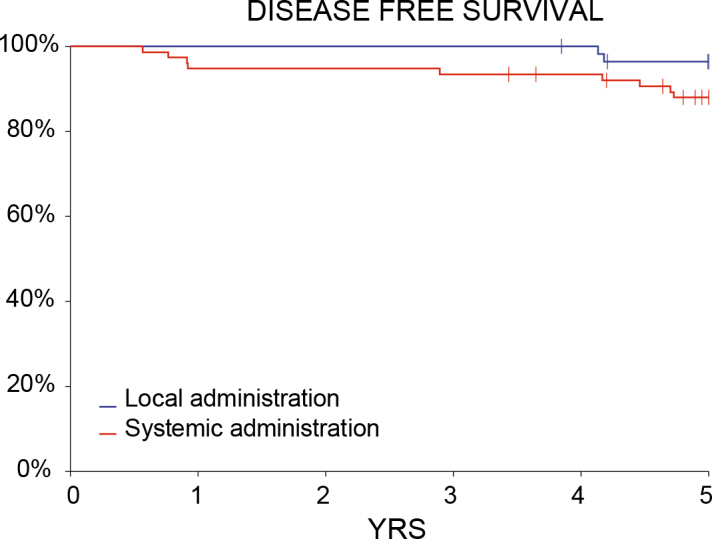
Disease-free survival (DFS) of nodules treated with local (intratumoral, IT) or systemic (intravenous, IV) drug administration. YRS = years

The treatment was equally effective whether the drug was injected IT or IV (p = 0.0877). Nevertheless, the long-term follow-up with DFS suggested better treatment outcomes after IT bleomycin injection in ECT (Figure 2).

### Toxicity

**FIGURE 3.**
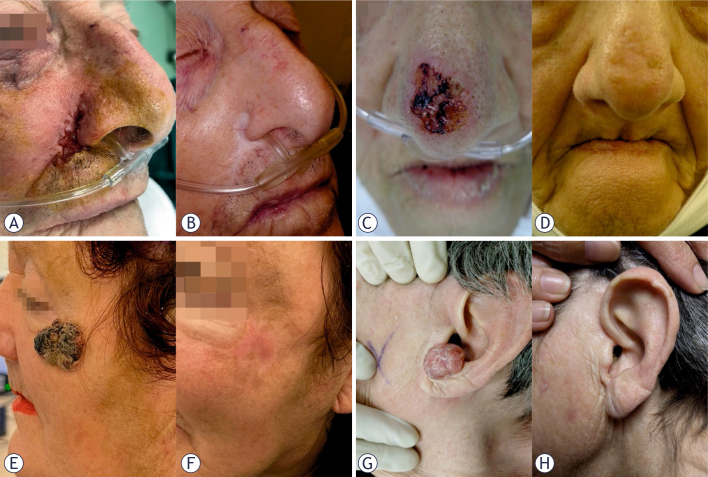
Treatment outcomes of patients treated with intravenous and intratumoral electrochemotherapy (ECT). The outcome is good in larger and smaller basal cell carcinoma (BCC) tumors. Patient No.1 **(A)** Recurrent BCC after multiple surgeries in a male, 82 years old patient. ECT performed under sedation and local anesthesia. Intravenous bleomycin 18200 IU. Needle row electrode; 11 pulses. Three cycles of ECT were performed (2 for the first treatment; 1 for recurrence after 2 years). **(B)** Result after 5 years of follow-up. Patient No. 2 **(C)** Primary BCC in a female, 86 years old patient with multiple severe comorbidities and non-suitable to standard treatments. One ECT session was performed under sedation and local anesthesia. Intralesional Bleomycin 1.5 ml (concentration 1 mg/ml). Needle row electrode; 7 pulses. **(D)** Result after 5 years of follow-up. Patient No. 3 **(E)** Primary BCC in a female, 69 years old patient. Patient refused standard treatments. One ECT session was performed in general anesthesia with laryngeal mask. Intravenous bleomycin 18200 IU; Hexagonal electrode; 9 pulses. **(F)** Result after 5 years of follow-up. Patient No.4 **(G)** Primary BCC in a female, 88 years old patient with severe comorbidities, Alzheimer’s disease and non-suitable to standard treatments. One ECT session was performed in general anesthesia with laryngeal mask. Intralesional bleomycin 1.5 ml (concentration 1 mg/ml); Finger electrode; 10 pulses. **(H)** Result after 5 years of follow-up.

ECT treatment was well tolerated, and 127 patients (96%) expressed readiness to undergo repeated treatment if necessary. This finding indicates high patient satisfaction with the tolerability and efficacy of the therapy, along with the absence of significant disfigurement or dysfunction (Figure 3). Among 36 patients (27%) who presented with an ulcerated lesion prior to ECT, only 14 (11%) exhibited persistent ulceration at the first follow-up after one month, which subsequently resolved completely upon healing. In 10 patients (8%), light hyperpigmentation remained after healing (Table 4).

**TABLE 4.** Symptoms and side effects per patient: comparison between pre-ECT values and 1-month post-ECT values

	Pre-ECT	1-month post-ECT
Nausea	1 (1%)	0 (0%)
Flu	0 (0%)	0 (0%)
Suppuration	1 (1%)	2 (1%)
Hyperpigmentation	1 (1%)	10 (8%)
Ulceration	36 (27%)	14 (11%)
Crust	0 (0%)	2 (1%)
Edema/rush	0 (0%)	3 (2%)

1 ECT = electrochemotherapy

**FIGURE 4.**
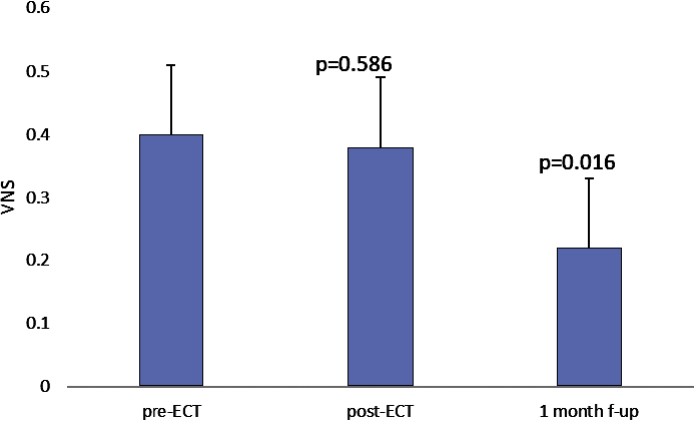
Pain intensity (visual numeric scale, VNS) preelectrochemotherapy (pre-ECT), immediately post-ECT and 1 month after ECT (mean and standard error).

Pain assessment using VNS showed no statistically significant difference between pre-ECT (0.4 ± 1.1, range 0 to 7) and immediately post-ECT (0.38 ± 1.17, range 0 to 8) evaluation (p = 0.586). However, a comparison of the pain scale at the first follow-up visit at 1 month (0.22 ± 1.12 range 0 to 9) with the pre-ECT evaluation revealed a statistically significant decrease (p = 0.016) (Figure 4). Additionally, two cases of skin rash and one case of facial edema were reported at the first follow-up, both of which resolved completely at subsequent visits.

### Short-term response: first-year follow-up

All patients achieved a complete response after 1 to 3 ECT sessions. In 127 patients (96%), a complete response was obtained after a single session. Due to a partial response, two sessions were required for four patients (3%), and three sessions were needed for one patient (1%). All retreatments were administered within 7 months of the initial session and were performed at 2, 4, 5, and 7 months (with two cases at 7 months).

During the first year of follow-up, BCC recurrence was observed in four patients (3%) between seven and eleven months after achieving a complete response to the initial treatment. Among them, two patients had recurrent BCC previously treated with surgical excision, while the remaining two had primary lesions. The tumor sizes ranged from 12 mm to 30 mm, with anatomical distribution located on the nose, temporal region, ear and multiple sites on the face. The recurrences were managed with further ECT in two patients, surgery in one patient and a combination of both ECT and surgery in one patient. Following the treatment of recurrence, all patients remained disease free until the end of the five-year follow-up period (Table 5).

**TABLE 5.** Characteristics of the patients who experienced recurrence during the first year of follow-up

Pt	Age	Primary	Previous therapy	Localization	Size (cm)	Time recurrence (months)	Treatment of the recurrence	Status at 5 years of follow-up
1	52	Recurrent	Surgery	Nasal ala	1.2	7	ECT	NED
2	73	Primary	No	Temporal region	1.5	10	Surgery	NED
3	65	Recurrent	Surgery	Ear	1.8	11	ECT, surgery	NED
4	52	Primary	No	Multiple (face)	3.0	11	ECT	NED

1 ECT = electrochemotherapy; NED = no evidence of disease; Pt = patient

### Long-term results

BCC recurrence was observed in 7 patients (5%) after the first year and throughout the subsequent four-year follow-up period. One patient had a recurrence at 2.9 years, while the remaining 6 developed a recurrence at least 4 years after ECT (median 4.4 years, range 4.1–4.7 years). Among these patients, 2 patients underwent surgical intervention, 2 were retreated with ECT, 1 received radiotherapy, and 2 declined further treatment. By the end of follow-up, 4 patients who underwent surgery or ECT were disease free, whereas 3 patients remained alive with disease. The locations of the lesions included 5 cases in the nasal region, 1 in the scalp region and 1 in the temporal region. The mean age of patients with long-term recurrence was 73 ± 10 years. A detailed summary of these patients is provided in Table 6.

**TABLE 6.** Characteristics of the recurrent patients

Pt	Age	Primary	Previous therapy	Localization	Size (cm)	Time recurrence (years)	Treatment of the recurrence	Status at 5 years of follow-up
1	82	Recurrent	Surgery	Nasal ala	2.5	2.9	ECT	NED
2	60	Primary	No	Temporal region	1.8	4.1	Surgery	NED
3	89	Recurrent	No	Nasal ala	1.7	4.7	No	AWD
4	84	Recurrent	No	Nasolabial fold	3.0	4.7	RT	AWD
5	68	Primary	Surgery	Tip of the nose	1.0	4.5	ECT	NED
6	74	Recurrent	Surgery	Nasolabial fold	1.6	4.2	No	AWD
7	72	Recurrent	Surgery +RT	Scalp	1.5	4.2	Surgery	NED

1 AWD = alive with disease; ECT = electrochemotherapy; NED = no evidence of disease; PT = patient; RT = radiotherapy

### Disease-free survival (DFS)

**FIGURE 5.**
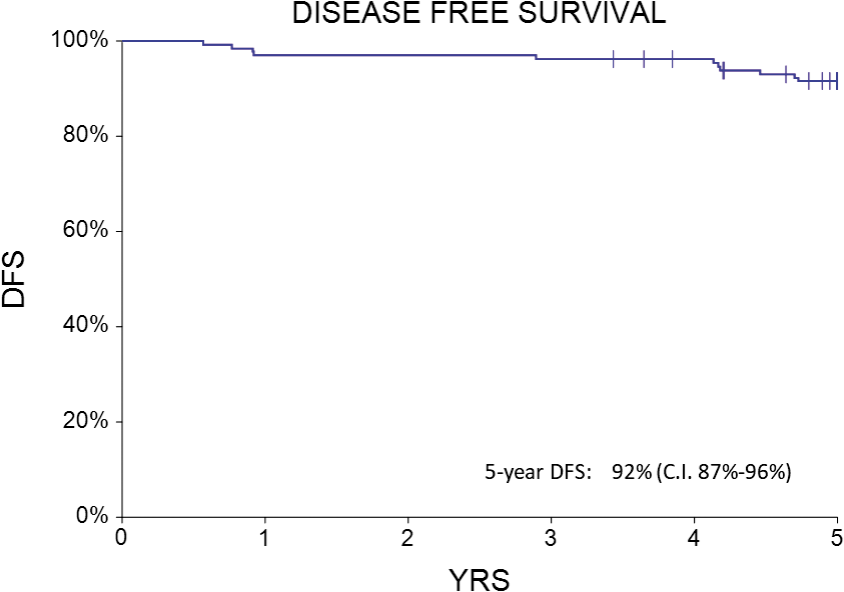
Kaplan-Meier survival curves for the whole population. DFS = disease free survival

**FIGURE 6.**
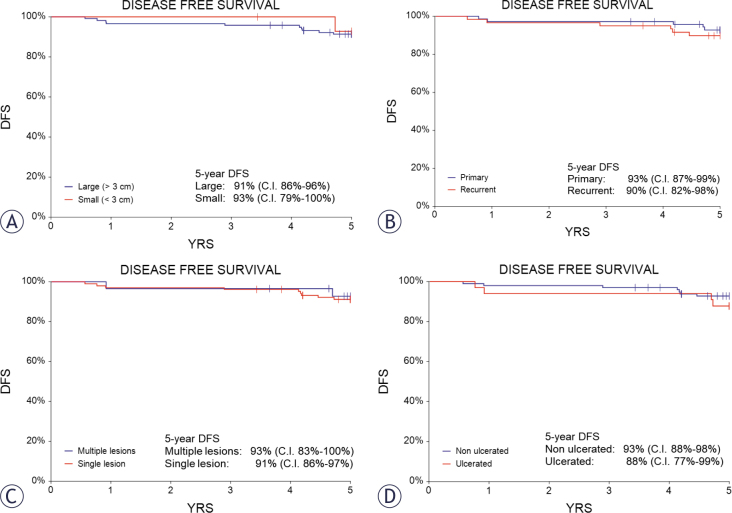
Kaplan-Meier survival **(A)** of patients with small (< 3 cm) vs large (> 3 cm) lesions **(B)**, patients with primary vs recurrent lesions **(C)**, patients with single vs multiple lesions **(D)**, and patients with ulcerated vs nonulcerated lesions. The differences were not significant. DFS = disease free survival; YRS = years

Over the five-year period following ECT treatment, patients were monitored for recurrence and DFS. At three years, the DFS rate was 96% (confidence interval [C.I.] 95%: 93%–99%), whereas at five years, it was 92% (C.I. 95%: 87%–96%) (Figure 5). DFS was slightly greater in primary lesions than in recurrent lesions, as well as in nonulcerated and small lesions, as illustrated in the Kaplan–Meier survival curves in Figure 6.

Recurrences were managed according to clinical indications and/or patient preferences. By the end of the five-year follow-up period, three patients (2%) were alive with disease, and 124 patients (98%) were disease free.

## Discussion

ECT has been established across various clinical settings as a highly effective treatment for cutaneous and subcutaneous tumors, regardless of histological type.^13,17,25^ Specifically, in the treatment of head and neck skin malignancies, ECT not only ensures effective oncologic control but also preserves aesthetic and functional outcomes. Consequently, it may represent a feasible alternative to surgery, particularly in cases where surgery is contraindicated or considered inappropriate.^14,15^

Although there are extensive data on the efficacy of ECT in the treatment of skin cancer, evidence on long-term control remains limited, particularly for BCC, which can recur years after treatment, especially in high-risk areas.^4,26^ Recently, a randomized control trial comparing the long-term outcomes of primary BCCs treated with ECT and surgery was published.^12^ The results of this study showed that, at the five-year follow-up, the recurrence rate in the surgical arm (1.9%) was comparable to that in the ECT arm (7.5%), with no statistically significant difference between the two groups (p = 0.33). The study results suggest that ECT is a durable treatment option for primary BCC and achieves similar long-term efficacy to surgery. These results are consistent with our findings, which demonstrated a five-year DFS rate of 92%. However, these data should be interpreted in the context of our cohort, which included patients with both primary and recurrent BCC. Furthermore, the treatment was administered exclusively to patients with BCCs in the head and neck region, which is known to comprise high-risk areas for BCC recurrence.^27^

Importantly, 45% of the BCC patients treated in our study were recurrences, which is generally associated with a poorer response to any treatment and presents a greater therapeutic challenge. Kaplan-Meier survival analysis showed that patients with primary lesions had slightly better DFS than those with recurrent lesions. Nevertheless, the five-year DFS rates for recurrent and primary BCC patients were 90% and 93%, respectively. The favorable long-term response to ECT in both recurrent and primary tumors can be attributed to the multifaceted mechanism of ECT. In addition to its direct antitumor effect, ECT also activates a local immune response, primarily through increased release of tumor antigens.^19,28,29^ This mechanism is particularly relevant in the treatment of BCCs, as these tumors have a high mutational burden.^30^ Consequently, the large amount of tumor antigens from necrotic BCCs following ECT stimulates the local immune response, which may further contribute to sustained disease control.

The long-term responses of small, nonulcerated lesions and large, ulcerated lesions did not differ significantly. However, nonulcerated and smaller lesions tend toward a greater likelihood of sustained disease control after ECT. This finding underscores the importance of patient and tumor selection when considering ECT and highlights its efficacy, particularly in well-defined, localized tumors.

Similarly, there was no statistically significant difference in response to ECT depending on whether bleomycin was administered intratumorally or systemically. However, the slightly attenuated response observed after systemic administration raises important clinical considerations. According to standard operating procedures for ECT, the route of administration should not affect the efficacy of the treatment.^17,22,23^ Nevertheless, vascularization plays an important role in therapeutic outcomes, as it affects the pharmacokinetics of bleomycin and thus the efficacy of ECT.^31^ The scarring process after previous treatments leads to vasculature remodeling within and around the tumor, which can influence the distribution of bleomycin by altering blood flow, perfusion and penetration of the drug into the tumor microenvironment. Therefore, it is possible that systemic administration of bleomycin could lead to poorer disease control in the case of relapse. However, further studies in a larger cohort of patients with histologically diverse tumors are needed to substantiate this hypothesis.

In our study, the majority of tumors treated with ECT were located in the H-zone (central face, eyelid, eyebrow, nose, lips, chin, ear, and periauricular regions), an area associated not only with a higher risk of recurrence but also with significant esthetic and functional effects of treatment.^26^ As far as the subjective assessment of patients is concerned, the high acceptance of ECT is evident, as 96% of patients stated that they would be willing to undergo treatment again if necessary. This is consistent with previous studies highlighting the favorable safety profile and low morbidity of ECT.^13,17^ The most common adverse effects were transient ulceration (11%) and mild hyperpigmentation (8%), both of which resolved over time without significant functional or aesthetic consequences. The minimal impact on pain perception, as shown by the stable VNS scores before and after treatment, also supports the tolerability of ECT. The safety and low morbidity profile are particularly important because 74% of our patients were over 65 years of age. This finding is in concordance with previous studies that emphasized the suitability of ECT for older patients with comorbidities, as standard treatments may represent a greater therapeutic burden in an older population.^9,10,32^

Our results regarding long-term recurrence rates are comparable to those of Mohs micrographic surgery, which is considered the gold standard for the treatment of high-risk facial BCCs, due to its high cure rate and tissue-sparing benefits. For example, a study that included 2,203 high-risk BCCs from the Danish Registry for Mohs Surgery reported a 5-year overall recurrence rate of 3.1% for primary BCCs and 5.3% for recurrent BCCs following Mohs micrographic surgery.^33^ However, one of the main advantages of ECT is that it can be repeated safely and without significant side effects. In our study, ECT was repeated in 2 out of the 7 patients who experienced recurrence.

Although our study supports the efficacy of ECT, it is important to recognize its limitations. A potential bias is that the response to treatment was evaluated solely by clinical assessment, without previous histologic or cytologic biopsies. This limitation is particularly relevant, as a previous biopsy might have identified a recurrence before it was clinically evident. Furthermore, the lack of direct comparisons with surgery or radiotherapy limits the assessment of the relative efficacy of ECT. Future prospective randomized trials with larger cohorts are needed to further clarify the role of ECT within the broader treatment algorithm for BCC, particularly in high-risk and recurrent cases.

In conclusion, our results support the role of ECT as a valuable alternative for the treatment of BCC in challenging anatomical locations. The high DFS rates, favorable safety profile and patient acceptance suggest that ECT can be integrated into clinical practice as a primary or adjunctive treatment modality. With advances in electroporation technology and drug delivery, further refinements in treatment protocols may enhance the therapeutic potential of ECT and solidify its position in the multidisciplinary treatment of BCC.

## References

[1] Levell NJ, Igali L, Wright KA, Greenberg DC. (2013). Basal cell carcinoma epidemiology in the UK: the elephant in the room. Clin Exp Dermatol.

[2] Heath MS, Bar A. (2023). Basal cell carcinoma. Dermatol Clin.

[3] Gordon R. (2013). Skin cancer: an overview of epidemiology and risk factors. Sem Oncol Nursing.

[4] Kim JYS, Kozlow JH, Mittal B, Kozlow JH, Mittal B, Moyer J (2018). Guidelines of care for the management of basal cell carcinoma. J Am Acad Dermatol.

[5] Cameron MC, Lee E, Hibler BP, Barker CA, Mori S, Cordova M (2019). Basal cell carcinoma. J Am Acad Dermatol.

[6] Peris K, Fargnoli MC, Kaufmann R, Arenberger P, Bastholt L, Seguin NB (2023). European consensus-based interdisciplinary guideline for diagnosis and treatment of basal cell carcinoma – update 2023. Eur J Cancer.

[7] Lyons P, Kennedy A, Clover AJP. (2021). Electrochemotherapy and basal cell carcinomas: first-time appraisal of the efficacy of electrochemotherapy on survivorship using FACE-Q. JPRAS Open.

[8] Russano F, Brugnolo D, Bisetto G, Del Fiore P, Rastrelli M, Mocellin S (2024). Electrochemotherapy treatment in a patient with an extended basal cell carcinoma of the face: a case report. JPM.

[9] Carpenè S, Silvestri B, Bertinazzi M, Armato E, Amadori M, Spinato R (2024). Electrochemotherapy as adjuvant treatment in a sinonasal mucosal melanoma in elderly patient: a case report. Eur Arch Otorhinolaryngol.

[10] Sersa G, Mascherini M, Di Prata C, Odili J, de Terlizzi F, McKenzie GAG (2021). Outcomes of older adults aged 90 and over with cutaneous malignancies after electrochemotherapy with bleomycin: a matched cohort analysis from the InspECT registry. Eur J Surg Oncol.

[11] Mir LM, Glass LF, Sersa G, Teissié J, Domenge C, Miklavcic D (1998). Effective treatment of cutaneous and subcutaneous malignant tumours by electrochemotherapy. Br J Cancer.

[12] Clover AJP, Salwa SP, Bourke MG, McKiernan J, Forde PF, O’Sullivan ST (2020). Electrochemotherapy for the treatment of primary basal cell carcinoma; a randomised control trial comparing electrochemotherapy and surgery with five year follow up. Eur J Surg Oncol.

[13] Clover AJP, de Terlizzi F, Bertino G, Curatolo P, Odili J, Campana LG (2020). Electrochemotherapy in the treatment of cutaneous malignancy: outcomes and subgroup analysis from the cumulative results from the pan-European International Network for Sharing Practice in Electrochemotherapy database for 2482 lesions in 987 patients (2008–2019). Eur J Cancer.

[14] Bertino G, Sersa G, De Terlizzi F, Occhini A, Plaschke CC, Groselj A (2016). European Research on Electrochemotherapy in Head and Neck Cancer (EURECA) project: results of the treatment of skin cancer. Eur J Cancer.

[15] Bertino G, Muir T, Odili J, Groselj A, Marconato R, Curatolo P (2022). the InspECT BCC Working Group. Treatment of basal cell carcinoma with electrochemotherapy: insights from the InspECT Registry (2008–2019). Curr Oncol.

[16] Sersa G, Miklavcic D, Cemazar M, Rudolf Z, Pucihar G, Snoj M. (2008). Electrochemotherapy in treatment of tumours. Eur J Surg Oncol.

[17] Marty M, Sersa G, Garbay JR, Gehl J, Collins CG, Snoj M (2006). Electrochemotherapy – an easy, highly effective and safe treatment of cutaneous and subcutaneous metastases: results of ESOPE (European Standard Operating Procedures of Electrochemotherapy) study. Eur J Cancer Suppl.

[18] Plaschke CC, Bertino G, McCaul JA, Grau JJ, de Bree R, Sersa G (2017). European Research on Electrochemotherapy in Head and Neck Cancer (EURECA) project: results from the treatment of mucosal cancers. Eur J Cancer.

[19] Sersa G, Ursic K, Cemazar M, Heller R, Bosnjak M, Campana LG. (2021). Biological factors of the tumour response to electrochemotherapy: review of the evidence and a research roadmap. Eur J Surg Oncol.

[20] Sersa G, Jarm T, Kotnik T, Coer A, Podkrajsek M, Sentjurc M (2008). Vascular disrupting action of electroporation and electrochemotherapy with bleomycin in murine sarcoma. Br J Cancer.

[21] Jarm T, Cemazar M, Miklavcic D, Sersa G. (2010). Antivascular effects of electrochemotherapy: implications in treatment of bleeding metastases. Expert Rev Anticancer Ther.

[22] Mir LM, Gehl J, Sersa G, Collins CG, Garbay JR, Billard V (2006). Standard operating procedures of the electrochemotherapy: Instructions for the use of bleomycin or cisplatin administered either systemically or locally and electric pulses delivered by the CliniporatorTM by means of invasive or non-invasive electrodes. Eur J Cancer Suppl.

[23] Gehl J, Sersa G, Matthiessen LW, Muir T, Soden D, Occhini A (2018). Updated standard operating procedures for electrochemotherapy of cutaneous tumours and skin metastases. Acta Oncol.

[24] Eisenhauer EA, Therasse P, Bogaerts J, Schwartz LH, Sargent D, Ford R (2009). New response evaluation criteria in solid tumours: Revised RECIST Guideline (version 1.1). Eur J Cancer.

[25] Bertino G, Groselj A, Campana LG, Kunte C, Schepler H, Gehl J (2022). Electrochemotherapy for the treatment of cutaneous squamous cell carcinoma: the INSPECT experience (2008-2020). Front Oncol.

[26] Peris K, Fargnoli MC, Garbe C, Kaufmann R, Bastholt L, Seguin NB (2019). Diagnosis and treatment of basal cell carcinoma: European consensusbased interdisciplinary guidelines. Eur J Cancer.

[27] Strom TJ, Caudell JJ, Harrison LB. (2016). Management of BCC and SCC of the head and neck. Cancer Control.

[28] Justesen TF, Orhan A, Raskov H, Nolsoe C, Gögenur I. (2022). Electroporation and immunotherapy – unleashing the abscopal effect. Cancers.

[29] Ferioli M, Perrone AM, De Iaco P, Zamfir AA, Ravegnini G, Buwenge M (2024). Clinical insights and future prospects: a comprehensive narrative review on immunomodulation induced by electrochemotherapy. Current Oncol.

[30] Jayaraman SS, Rayhan DJ, Hazany S, Kolodney MS. (2014). Mutational landscape of basal cell carcinomas by whole-exome sequencing. J Invest Dermatol.

[31] Groselj A, Kranjc S, Bosnjak M, Krzan M, Kosjek T, Prevc A (2018). Vascularization of the tumours affects the pharmacokinetics of bleomycin and the effectiveness of electrochemotherapy. Basic Clin Pharmacol Toxicol.

[32] Jamsek C, Sersa G, Bosnjak M, Groselj A. (2020). Long term response of electrochemotherapy with reduced dose of bleomycin in elderly patients with head and neck non-melanoma skin cancer. Radiol Oncol.

[33] Andersen YMF, Karmisholt K, Høgsberg T, Nielsen ML, Brosbøl A, Stave S (2025). 10 years of Mohs micrographic surgery in Denmark: results from a nationwide cohort. Acta Derm Venereol.

